# Stabilized mosaic single-cell data integration using unshared features

**DOI:** 10.1038/s41587-023-01766-z

**Published:** 2023-05-25

**Authors:** Shila Ghazanfar, Carolina Guibentif, John C. Marioni

**Affiliations:** 1 Cancer Research UK Cambridge Institute, University of Cambridge, Cambridge, UK; 2 European Molecular Biology Laboratory, European Bioinformatics Institute, Wellcome Genome Campus, Cambridge, UK; 3 School of Mathematics and Statistics, The University of Sydney, Camperdown, New South Wales Australia; 4 Charles Perkins Centre, The University of Sydney, Camperdown, New South Wales Australia; 5 Sahlgrenska Center for Cancer Research, Inst. Biomedicine, Dept. Microbiology and Immunology, University of Gothenburg, Gothenburg, Sweden; 6 Wellcome Sanger Institute, Wellcome Genome Campus, Cambridge, UK

**Keywords:** Data integration, Computational models

## Abstract

Currently available single-cell omics technologies capture many unique features with different biological information content. Data integration aims to place cells, captured with different technologies, onto a common embedding to facilitate downstream analytical tasks. Current horizontal data integration techniques use a set of common features, thereby ignoring non-overlapping features and losing information. Here we introduce StabMap, a mosaic data integration technique that stabilizes mapping of single-cell data by exploiting the non-overlapping features. StabMap first infers a mosaic data topology based on shared features, then projects all cells onto supervised or unsupervised reference coordinates by traversing shortest paths along the topology. We show that StabMap performs well in various simulation contexts, facilitates ‘multi-hop’ mosaic data integration where some datasets do not share any features and enables the use of spatial gene expression features for mapping dissociated single-cell data onto a spatial transcriptomic reference.

## Abstract (web summary)

Mosaic data integration is improved by capturing features that do not overlap between datasets.

## Main

Large-scale efforts to build transcriptional maps of tissues at cellular resolution have revealed many biological insights and provided reference maps that can be used to further interrogate biological systems^1,2^. Simultaneous technological advances have led to the generation of datasets that capture multiple distinct types of molecular information, for example, cellular indexing of transcriptomes and epitopes (CITE-seq) captures RNA expression and cell surface protein abundance^3^, and 10x Genomics Multiome captures RNA expression alongside DNA fragments associated with regions of open chromatin^4^. Consequently, data integration has emerged as a key challenge for consolidating and profiting from such rich resources^5^, with the task of integrating diverse molecular assays being known as ‘mosaic data integration’^6^, as distinct from horizontal data integration where multiple sets of cells are measured using the same features, and vertical data integration where multiple sets of features are measured on the same population of cells. At present, many methods for mosaic data integration are typically limited to using the set of overlapping features between modalities^7,8^.

However, as the number and complexity of single-cell datasets increase, there is a growing need to develop techniques specifically designed to perform mosaic data integration^9,10^. Some existing approaches designed to tackle this problem include UINMF^11^, which introduces a latent metagene matrix in the factorization problem, and MultiMAP^12^, a graph-based method that assumes a uniform distribution of cells across a latent manifold structure fitted using an optimization approach. A critical limitation of both approaches, however, is the requirement that there exist at least some core features that are shared across all datasets, resulting in analysts needing to compromise on input datasets, or making the ‘central dogma assumption’, that is, matching features between different omics modalities based on corresponding DNA–RNA–protein sequences. Moreover, while MultiMAP includes a tuning parameter to prioritize certain datasets, neither approach offers a supervised mode that takes into account a priori cell labels.

Additional approaches, such as Cobolt^13^ and MultiVI^14^, aim to capitalize on jointly profiled multiomics technologies, most notably single-cell RNA sequencing (scRNA-seq) and single-cell assay for transposase-accessible chromatin using sequencing (ATAC-seq), by integrating these with existing single-modality datasets. These approaches treat the multiomic dataset as the ‘bridge’ to enable joint embedding of all single omic and multiomic data, thereby enabling multi-hop mosaic data integration. While effective at the specific RNA + ATAC integration task, these methods currently lack flexibility and generalizability to incorporate additional datasets. Other approaches, such as SingleCellFusion^15^, instead rely on relationships between features, for example, transcriptomic and epigenomic, to jointly embed distinct single modalities into a joint space. For spatially resolved single-cell gene expression data, approaches such as SPaGE^16^ and Tangram^17^ accurately map dissociated scRNA-seq data onto spatial coordinates; however, they are unable to benefit jointly from the (1) additional features present in scRNA-seq data and (2) robust neighborhood-aware spatial features extracted from spatial omics data.

In this Article, we introduce StabMap, a data integration technique designed specifically for mosaic data integration tasks. StabMap projects all cells onto supervised or unsupervised reference coordinates using all available features regardless of overlap with other datasets, instead relying on traversal along the mosaic data topology (MDT). By using multiple simulation scenarios and by exploring spatially resolved transcriptomic data, we show that StabMap performs well, in particular in the presence of very few overlapping features. Additionally, we demonstrate StabMap’s ability to perform multi-hop mosaic data integration and reveal biological insights into the role of Brachyury in early mouse organogenesis.

## Results

### StabMap: stabilized mapping for mosaic single-cell data integration

**Fig. 1:**
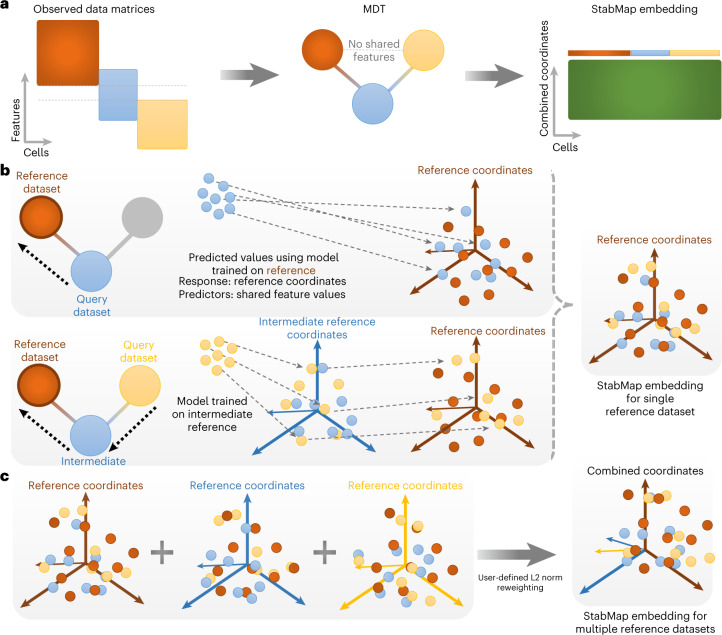
StabMap method overview. **a**, Example mosaic data integration displaying observed data matrices with varying overlap of features among the datasets. Datasets are summarized using the MDT. Cells are then projected onto the common StabMap embedding across all cells. **b**, Cells from all datasets are projected onto the reference space (dark red) by traversing the shortest paths along the MDT. Blue cells are projected directly onto the reference space, whereas yellow cells are first projected onto the space defined by the blue cells, followed by projection to the dark-red space. All cells are then combined to yield the common StabMap embedding. **c**, The process described in **b** is performed for various selected reference datasets (default = all), followed by L2-norm reweighting provided by the user (default = equal weight). These reweighted embeddings are then concatenated to form the StabMap embedding for multiple reference datasets, and can be used for further downstream analysis tasks.

The input to StabMap is a set of single-cell data matrices, one or more of which can be identified as reference datasets (default all), and an optional set of discrete cell labels. From this data structure StabMap extracts the MDT, a network with nodes corresponding to each given dataset, and edges between nodes, weighted by the absolute number of shared features between the datasets (Fig. 1a). StabMap requires only that the MDT be a connected network, that is, that there be a way to draw a path from each node to every other node. For the selected reference dataset, *R*, a supervised (linear discriminant (LD) analysis, if labels provided) or unsupervised (principal component (PC) analysis) dimensionality reduction algorithm is employed, generating a feature loading matrix for the discriminants or components. Alternatively, if a lower-dimensional embedding already exists for this reference data, for example, resulting from application of a vertical integration method such as MOFA^18^ or Seurat v4 (ref. ^19^), it can be provided by the user. This is performed using all features available for the reference dataset. Then, for each non-reference dataset, *D*, the shortest path is identified between *R* and *D* along the MDT. If there is a direct link between *R* and *D*, a multivariable linear model is fitted to estimate the PC and/or LD scores, with predictor variables corresponding to the shared features between datasets *R* and *D*. If there is no direct link between *R* and *D*, StabMap will construct a sequence of mappings between features traversing the shortest path between *R* and *D* along the MDT by iteratively predicting the scores of the reference dataset (Fig. 1b and Methods). In the case where multiple datasets are considered as reference datasets (by default all datasets are considered references), the process is repeated. All resulting embeddings are then reweighted (default equal weights) and concatenated to form a single low-dimensional matrix (Fig. 1c and Methods). The resulting StabMap embedding can be employed for further downstream analysis tasks, including batch correction, joint visualization, supervised and unsupervised machine learning tasks, differential abundance testing, and testing for and characterizing developmental trajectories.

By performing mosaic data integration using traversal along the MDT, and not relying on the features common to all datasets, StabMap unlocks the ability to perform multi-hop mosaic data integration, that is, integrating data where the intersection of features measured for all datasets is empty. Since StabMap results in a low-dimensional embedding common to all datasets, it can be combined with further downstream horizontal data integration tasks, such as mutual nearest neighbors^20^, Seurat^21^ and scMerge^22^, to adjust for any remaining batch effects.

### StabMap preserves cell–cell relationships in multiomic data

**Fig. 2:**
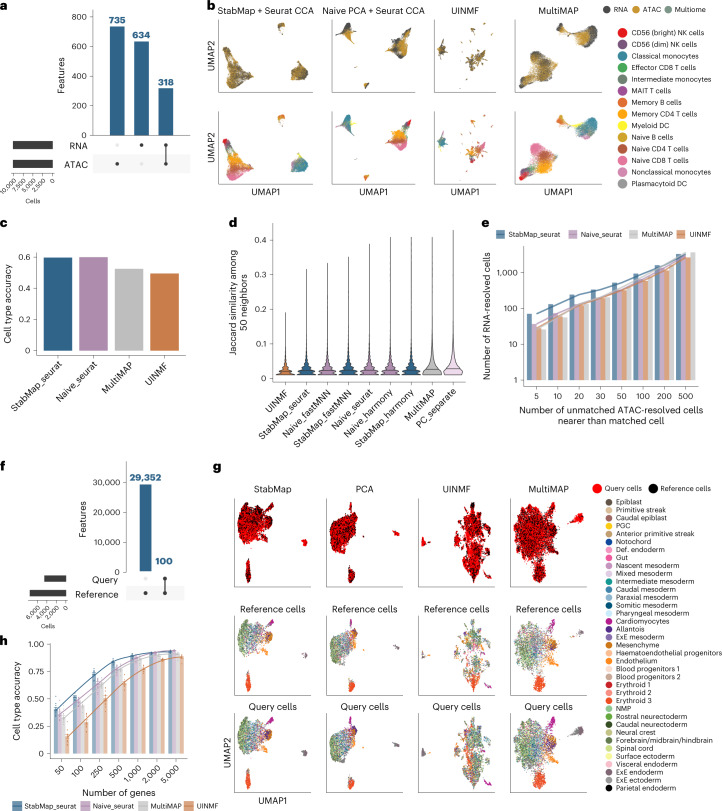
Mosaic data integration simulations using PBMC Multiome and Mouse Gastrulation Atlas data. **a**, UpSet plot of features shared between simulated RNA and ATAC modalities. ATAC peaks in promoter regions of genes are aligned with the genes in the RNA modality, resulting in 318 common features, 735 and 634 features distinct to the ATAC and RNA platforms, respectively. **b**, UMAP representations of RNA and ATAC modality cells for StabMap (first column), PCA, UINMF and MultiMAP (last column), colored by simulated modality (top row) and by cell type (bottom row). **c**, Bar plot of cell type classification accuracy predicting ATAC-resolved cell types using RNA-resolved cells as training data. **d**, Violin plots displaying Jaccard similarity among 50 neighbors for cells in each modality, where a higher value indicates a better preservation of neighborhood structure. **e**, Bar plot displaying the cumulative number of RNA-resolved cells, grouped by the number of unmatched ATAC-resolved cells found to be nearer than the matched ATAC-resolved cell. Ideally all RNA-resolved cells would be placed near their matching ATAC-resolved cells; therefore, more positive values indicate more cells nearer to their true matching cell and better quality of recapturing cell relationships. **f**, UpSet plot of features between simulated query and reference datasets for Mouse Gastrulation Atlas data. In this example the query dataset contains only 200 features, whereas the reference dataset contains those features along with 9,372 additional features. **g**, UMAP representations of Mouse Gastrulation Atlas data simulation scenario described in **f** using StabMap, PCA, MultiMAP and UINMF. The first row shows the query cells colored by cell type, the second row shows reference cells colored by cell type, and the third row shows query cells colored by cell type. **h**, Bar plot displaying the cell type classification accuracy of query cells for various methods, when the query set is restricted to different numbers of genes. Error bars represent mean ± standard error of the mean. Cell type classification is performed for all combinations of query and reference sample sets, totaling 12 repetitions. Def. endoderm = definitive endoderm. ExE mesoderm = extraembryonic mesoderm.

To investigate the performance of StabMap, we first constructed a simulation scenario using multiomics single-cell data, where chromatin accessibility and messenger RNA expression were measured in each of ~36,000 peripheral blood mononuclear cells (PBMCs)^23^. Using these data, we computationally created two single-cell datasets—one containing only the mRNA measurements and the other only the chromatin accessibility measurements—and assumed that the problem of interest was to combine these two datasets onto a common scaffold. We used all highly variable genes (HVGs) from the RNA modality, and all highly variable peaks from the ATAC modality, and considered the peaks associated with promoter regions of genes as common features (Fig. 2a).

Within this context, we compared StabMap’s performance with (1) a naive approach where PCA was applied only to overlapping features, (2) with UINMF and (3) with MultiMAP. In general, we observed reasonable mixing of the RNA- and ATAC-simulated cells with each other across all four computational approaches, as well as distinct separation of cell types (Fig. 2b). However, when assessing performance using more quantitative metrics, including the accuracy with which cell types could be predicted (when using the ATAC as the testing set and the RNA as the training set) and the preservation of the distances between cells in the common space, we noted more substantial differences (Methods and Fig. 2c–e). Specifically, we observed that, while StabMap generally performed well, the other methods (especially the naive PCA implementation and UINMF) had difficulty in accurately predicting cell type (Fig. 2c) and in preserving local neighborhood structure (Fig. 2e). Taken together, these results suggest that StabMap is well able to perform mosaic data integration.

### StabMap has superior performance with non-optimal features

**Extended Data Fig. 1:**
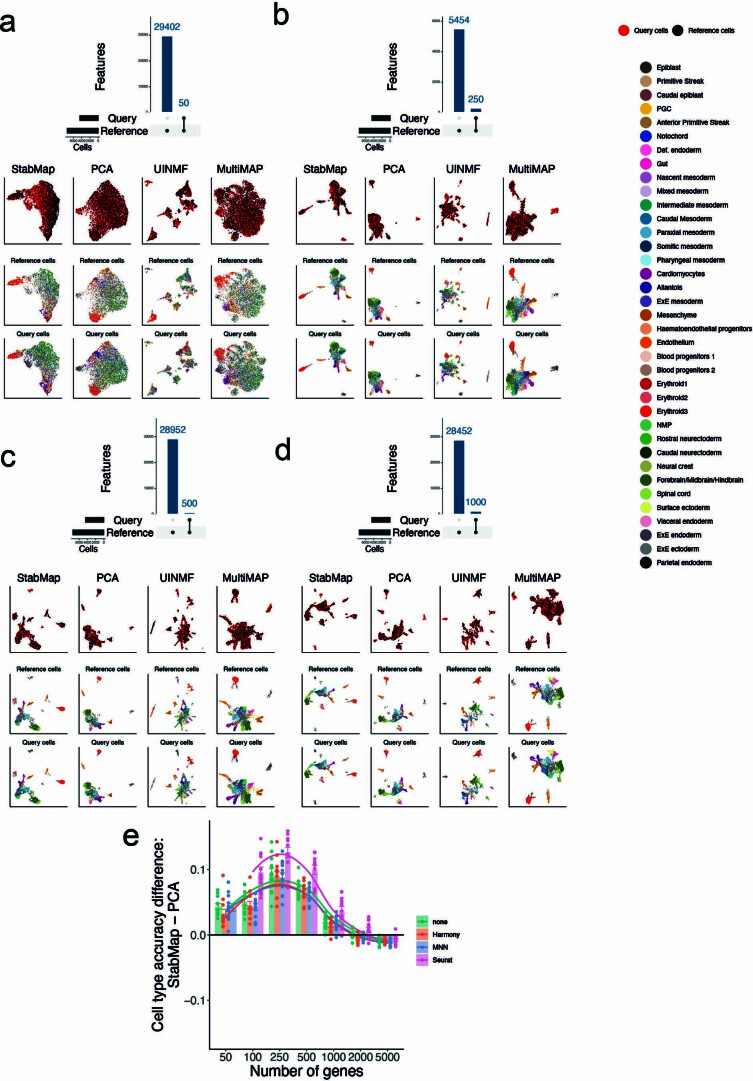
Comparison of StabMap using Mouse Gastrulation Atlas data. **a**. UpSet plot and UMAP representations of Mouse Gastrulation Atlas data simulation with 100 randomly selected features using StabMap, PCA, MultiMAP, and UINMF. First row shows the query cells coloured by simulated dataset, the second row shows reference cells coloured by cell type, and the third row shows query cells coloured by cell type. **b-d**. As in panel (a.) for 500, 1,000, randomly selected and all features respectively. **e**. Barplot displaying the difference in cell type prediction accuracy (y-axis) in the Mouse Gastrulation Data simulation, where data is integrated using StabMap or the naive PCA approach. StabMap displays a higher cell type accuracy for many choices of the number of genes (x-axis) for all choices of downstream horizontal integration (none, Harmony, Mutual Nearest Neighbours (MNN) and Seurat), and as the number of genes increases, this difference reduces closer to zero, indicating that the gain in accuracy is much more pronounced for smaller numbers of genes. Cell type classification is performed for all combinations of query and reference sample sets totalling 12 repetitions. Data are presented as mean values + /- SEM.

To further investigate the properties of StabMap, we used scRNA-seq data generated to study mouse gastrulation across entire embryos and at multiple timepoints^1^ in order to simulate a mosaic data integration task where the reference data contains an assay that captures the full transcriptome (that is, from scRNA-seq), and the query data contain only a subset of the available gene expression features (for example, as would be the case for technologies such as seqFISH^24^, MERFISH^25^, qPCR and so on). We considered the situation where the most informative features are not necessarily known a priori, and split the cells into two datasets, for which one was assumed to contain a small number of genes (*n* = 50, 100, 250, 500, 1,000, 2,000 and 5,000) randomly selected from among the HVGs in the reference data (Fig. 2f and Methods). We compared StabMap with UINMF, MultiMAP and PCA, and visually noted the decrease in structure apparent among the query cells in the common embedding for these other methods compared to StabMap (Fig. 2g and Extended Data Fig. 1). A common task when mapping a query dataset to a reference dataset is to predict the cell types of the query cells. Consequently, we assessed the quality of the data integration task by calculating the *k*-nearest neighbors cell type classification accuracy (Methods). We identified a much higher accuracy for StabMap, especially when very few features were captured in the simulated query datasets (Fig. 2h), independent of choice of downstream horizontal data integration (Extended Data Fig. 1e). Taken together, our results suggest that StabMap is effective at stabilizing mapping between datasets even when some of the datasets/modalities contain non-optimal features.

### Multi-hop mosaic data integration

**Fig. 3:**
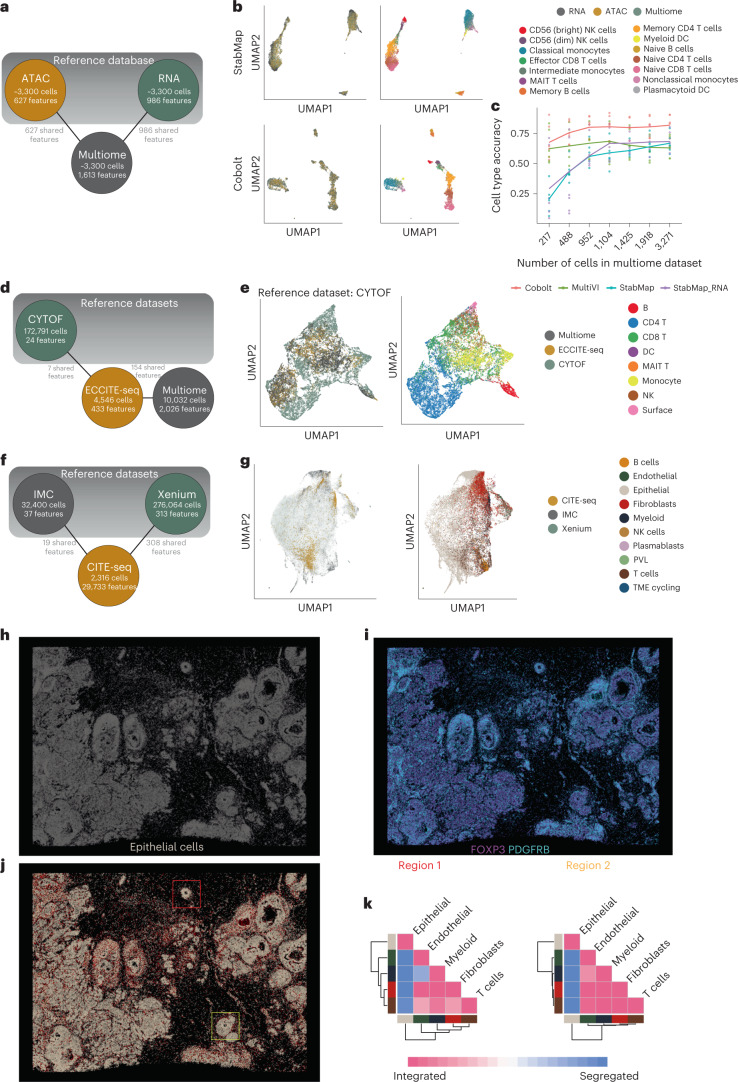
Multi-hop mosaic data integration simulation and real data analyses. **a**, Summary of mosaic data integration for PBMC Multiome simulation. Nodes present in the top shaded region are selected as reference datasets in the simulation. **b**, Joint two-dimensional embeddings generated using StabMap (first row, UMAP) and Cobolt (second row, UMAP), colored by simulated data type (left), and by cell type (right). **c**, Scatter plot displaying cell type accuracy (*y* axis) predicting ATAC-seq resolved cells using scRNA-seq-resolved cells as the training data, as the number of cells in the Multiome (*x* axis) increases. Each point corresponds to a simulation scenario and choice of multi-hop mosaic data integration method, including Cobolt, MultiVI, StabMap (default parameters) and StabMap_RNA (only RNA modality selected as reference). **d**, MDT of PBMC multiomics integration. Features are shared among the ECCITE-seq and CYTOF and Multiome datasets, respectively, but there are no shared features between the CYTOF and Multiome datasets. **e**, Joint UMAP embedding of multi-hop StabMap with CYTOF as the reference dataset, colored by data modality (left) and broad cell type (right). **f**, MDT of breast cancer spatial omics and multiomics integration. IMC and Xenium datasets are retained as reference datasets in this analysis. **g**, Joint UMAP embedding of StabMap colored by the data modality (left) and broad cell type (right). **h**, Spatial plot of Xenium-resolved cells in physical coordinates that are predicted to be epithelial using the IMC-resolved cells as training data. **i**, Spatial plot of Xenium-resolved cells in physical coordinates colored by imputed protein signal as measured from IMC-resolved data, for proteins PDGFRB (cyan) and FOXP3 (purple). **j**, Spatial plot of Xenium-resolved cells colored by predicted broad cell type using IMC-resolved cells as training data. Color legend is the same as in panel **g**. Two regions of interest are identified in red (region 1) and yellow (region 2) boxes, corresponding to a triple-positive receptor region and an invasive region, respectively. **k**, Cell–cell contact maps generated for the two regions according to broad cell type predicted value, indicating the degree of mixing of cells than expected by chance.

**Extended Data Fig. 2:**
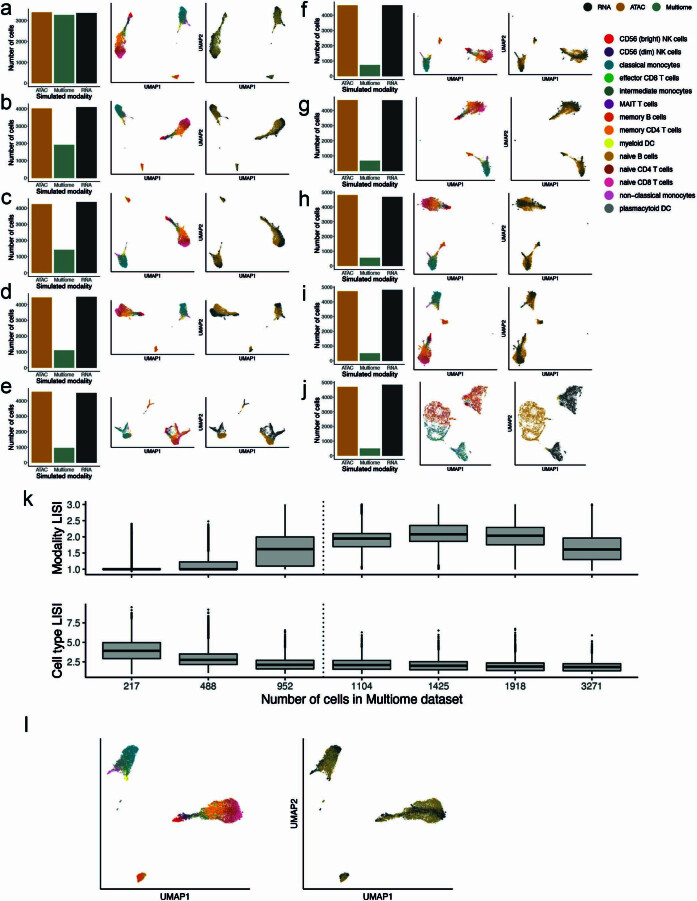
StabMap performance in 10X PBMC Multiome simulation. **a**. Number of 10X PBMC Multiome cells assigned to each simulated data type (left), joint UMAP generated using StabMap coloured by simulated data type (middle), and by cell type (right). **b-j**. As in panel (a.) for decreasing proportions of simulated Multiome cells. **k**. Local inverse Simpson indices (LISI) for simulated data type (top row) and for cell type (bottom row). LISI values are extracted for all integrated cells (n = 10,032). Each boxplot (median bar and whiskers to quartiles) corresponds to different choices of number of cells in the multiome dataset. The dotted line indicates approximately 1,000 cells in the multiome dataset, where LISI values appear to markedly shift from unfavourable to favourable integration. **l**. Joint UMAP embedding generated using StabMap in simulation as described in Fig. 3, with RNA dataset selected as reference, indicating a ‘multi-hop’ data integration.

Since StabMap relies on the MDT of the datasets, multiple datasets where some pairs of datasets do not share any features can be embedded into the same StabMap space. This contrasts with existing implementations of PCA, UINMF and MultiMAP, all of which require at least one feature to be shared across all datasets. While this is a major advantage of StabMap, we reasoned that its ability to perform multi-hop mosaic data integration would depend heavily on the quality of the input datasets. Consequently, we established how reliably StabMap was able to perform multi-hop mosaic data integration with differing levels of information content. Using the 10x Genomics PBMC Multiome data, we randomly split the cells equally into three simulated data types, RNA only, ATAC only and Multiome (Methods). We intentionally opted to not assign ATAC promoter peak IDs to gene names (that is, opting to not make the ‘central dogma assumption’), to replicate the multi-hop mosaic data integration task, such that there are no explicitly shared features between the RNA only and ATAC only datasets (Fig. 3a). We observed that StabMap successfully integrated these three datasets, with cells evenly distributed by data modality, and distinct cell type identities being clearly visible (Fig. 3b). We compared our multi-hop mosaic integration with two approaches specifically designed for multiomic data integration, Cobolt and MultiVI, and visually observed similar high-quality joint integration. We observed that Cobolt, a method designed specifically for integration of scRNA-seq and single-cell ATAC-seq data, performed consistently better in recapturing cell type labels (Fig. 3b,c). Since the most connected node in the MDT is the Multiome dataset, we next queried whether the quality of the StabMap embedding would deteriorate when fewer cells were present in this Multiome dataset. Indeed, we found that when fewer than ~1,000 cells were allocated to the Multiome dataset, the quality of the StabMap embedding was compromised, with poor local inverse Simpson’s index (LISI)^26^ values relative to modality and cell type (Extended Data Fig. 2a–k). In addition, we found the choice of reference dataset did not affect performance of StabMap (Extended Data Fig. 2l) such as the choice of RNA modality only as reference. When the ‘bridge’ datasets contained more than 1,000 cells we observed highly consistent performance, suggesting that multi-hop mosaic integration with StabMap is robust as long as a moderately sized bridge dataset is present.

**Extended Data Fig. 3:**
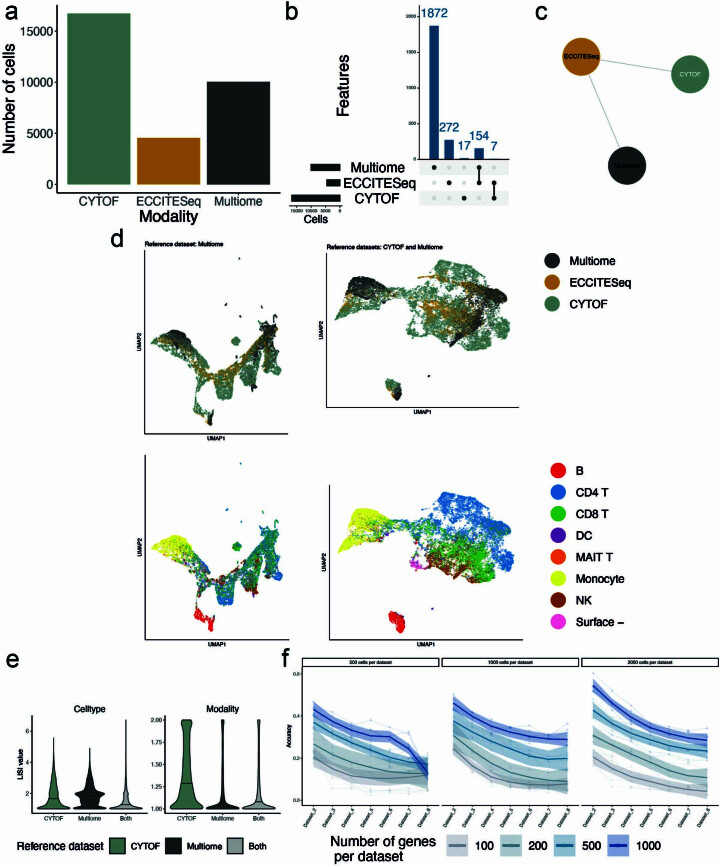
Multi-hop integration of PBMCs using StabMap. **a**. Number of cells present in CYTOF, ECCITE-Seq and Multiome PBMC datasets. **b**. UpSet plot of features shared among datasets, for example 7 proteins are measured in the CYTOF and ECCITESeq datasets, gene expression is measured for 154 genes in the Multiome and ECCITESeq datasets, while all other protein, RNA and chromatin accessibility features are distinct. **c**. Mosaic data topology of these datasets. Features are shared among the ECCITESeq and CYTOF and Multiome datasets respectively, but there are no shared features between the CYTOF and Multiome datasets. **d**. Joint UMAP embeddings of multi-hop Stabmap performed with reference dataset Multiome (left column) and both CYTOF and Multiome (right column), coloured by the data modality (top row) and broad cell type (bottom row). **e**. Violin plots of LISI values among CYTOF and Multiome cells for the three embeddings as in panel d. LISI values are calculated with reference to broad cell type (left), where low values are more desirable, and with reference to modality (right), where high values are considered more desirable. Overall we observe more desirable mixing of cells when using the CYTOF dataset as the reference for this scenario. **f**. Line plots indicating the preservation of biological signal across several steps of multi-hop mosaic data integration. Cells were randomly selected from the Mouse Gastrulation Dataset, and split into 8 distinct datasets (x-axis) with varying numbers of total cells per dataset n = 500, 1,000, 2,000 (panels). Then, varying numbers of features n = 100, 200, 500, 1,000 (lines in each plot) were retained from among the HVGs such that there was approximately 50% overlap of features between datasets 1 and 2, 2 and 3, and so on. As a result, any one dataset only shares features with its neighbouring dataset, representing an extreme task for multi-hop mosaic data integration. To assess quality, cell type accuracy was calculated with dataset 1 as the reference (y-axis), and we observe some decrease in mapping quality as the number of intermediate datasets increased, especially as fewer features were used. Ribbons represent 95% confidence intervals on generalised additive model smoothed curve.

To further examine the capabilities of StabMap, we performed a joint mapping spanning proteomics, transcriptomics and chromatin accessibility in PBMCs. We collected CyTOF^27^, ECCITE-seq^28^ and previously mentioned 10x Genomics Multiome data, and performed multi-hop mosaic integration using CyTOF and 10x Multiome as reference datasets (Fig. 3d,e, Extended Data Fig. 3a–d and Methods). We observe slightly better mixing when the CYTOF data are retained as the reference dataset (Extended Data Fig. 3e), which may be due to more comprehensive representation of cell type diversity, or the biological information retained in the protein features measured. In addition, we performed a joint mapping between spatial proteomics, single-cell multiomics and spatial transcriptomics. We collected imaging mass cytometry (IMC)^29^, CITE-seq^30^ and 10x Genomics Xenium^31^ data from breast tumor samples with positive HER2 status, and performed multi-hop mosaic integration using IMC and Xenium datasets as references (Fig. 3f,g). In doing so, we were able to extend the quality of the Xenium data by predicting the annotation of epithelial cells as curated in the IMC data (Fig. 3h) and impute the protein signal onto the Xenium-resolved tissue (Fig. 3i). In addition, our prediction of broad cell types as curated by the IMC-resolved data allowed us to predict cell types for the Xenium-resolved data, and use our previous statistical approach^7^ to build local cell–cell contact maps of distinct cell types (Fig. 3j,k). Focusing on a triple-positive receptor region (region 1) and an invasive region (region 2), we noted separation of epithelial cells from all other cell types, and observed a slightly higher degree of mixing of T cells with other non-epithelial cells in the invasive region 2 than expected by chance. Together, this mosaic data analysis shows the ability to harness the strengths of distinct datasets to lead to further understanding and hypothesis generation.

To further assess the capabilities of StabMap in multi-hop mosaic integration, we performed a simulation where we randomly selected cells from the Mouse Gastrulation Dataset, and split into eight distinct datasets that shared features sequentially, that is, Dataset *i* shared features only with Datasets *i*-1 or *i+*1 (Methods). As we varied the number of cells and HVGs per dataset, we observed better preservation of biological signal between Dataset 8 and Dataset 1 (Extended Data Fig. 3f) with inclusion of more informative features, and to a lesser extent with more cells per dataset. More generally, this suggests that multi-hop mosaic integration is robust to several datasets while feature quality remains high.

### Spatial mapping of mouse chimera identifies differences along major anatomical axis

**Fig. 4:**
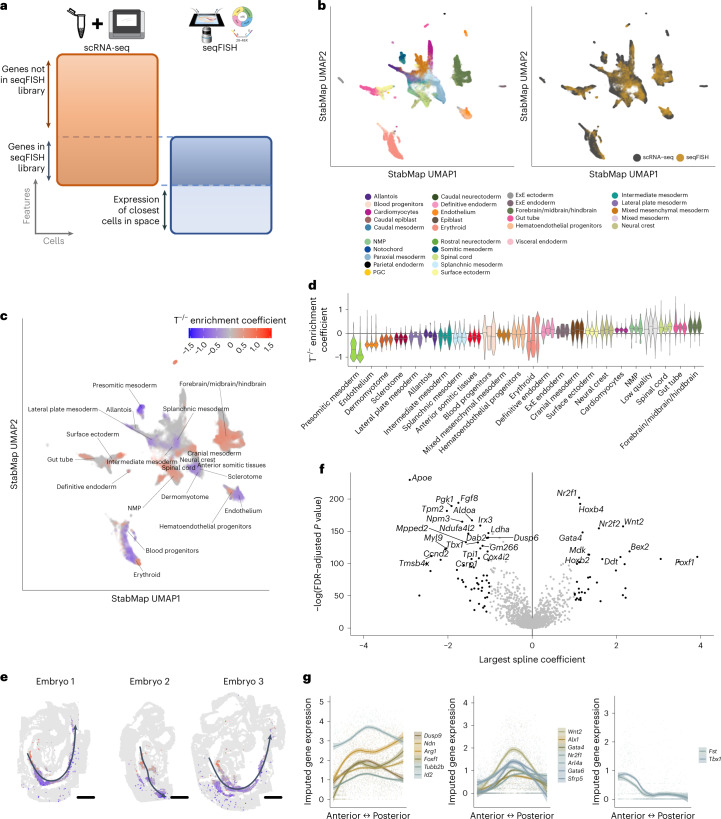
Integration of T-chimera and seqFISH data using StabMap with spatial neighbor feature extraction. **a**, Summary of mosaic data integration task and features used. Cells captured using scRNA-seq belonging to the E8.5 mouse gastrulation atlas^1^, WT/WT chimera^1^ and T^−/−^/WT chimera^32^. seqFISH cells are obtained from sagittal sections of three E8.5 embryos^7^. Features used for the scRNA-seq data are the union of the HVGs for each dataset. Features used for the seqFISH data are the gene expression of each cell, as well as the mean gene expression of the most proximal cells in space. **b**, UMAP plots displaying all cells after performing StabMap. Cells are colored by cell type (left) and by platform (right). **c**, UMAP plot of all seqFISH cells colored by local enrichment coefficient value of T^−/−^ enrichment test for statistically significant tests. **d**, Violin plots of T^−/−^ enrichment coefficients per embryo split by cell type. **e**, Spatial graphs of seqFISH embryos, with cells colored by T^−/−^ coefficients for cells assigned a splanchnic mesoderm identity. Curved lines are fitted principal curves associated with the AP axis along each embryo. Scale bar, 150 μm. **f**, Volcano plot showing value of largest magnitude spline coefficient (*x* axis) and −log(FDR-adjusted *P* value) for likelihood ratio test of splines model for splanchnic mesoderm (Methods). Top 30 highly ranked genes with large spline coefficients above a magnitude of 1 are labeled. **g**, Scatter plots and local mean expression ribbons of clustered genes showing distinct patterns of expression along the AP axis in splanchnic mesoderm. Bands represent 95% confidence for loess smoothed fit. ExE endoderm = extraembryonic endoderm, PGC = primordial germ cells.

A distinct advantage of mosaic data integration is the ability to integrate datasets where distinct features have been probed. An additional advantage is that the joint embedding can be used to facilitate downstream analyses, including differential abundance testing across experimental groups. To demonstrate this, we explored embryonic day (E)8.5 single-cell RNA-seq data from the mouse^1^, together with perturbation experiment data in the form of Brachyury (T) knockout T^−/−^/wild-type (WT) chimeras and control WT/WT chimeras collected at the same timepoint^32^. Chimeric embryos contain a mix of host (WT) cells and injected cells that are labeled with td-Tomato; the injected cells in the control chimera are WT, while the injected cells in the T^−/−^/WT chimeras lack a functional copy of Brachyury (T)^32^. We also considered single-cell resolution spatially resolved seqFISH data from a similar developmental timepoint^7^. For the scRNA-seq datasets we considered the union of HVGs, while for the seqFISH data we considered all 351 genes that were probed in the experiment. Additionally, for the seqFISH data, we extracted new features, corresponding to the mean expression of each gene among the immediate neighbors of each cell, thus providing information about each cell’s local, spatially resolved context (Fig. 4a and Methods). We used StabMap to jointly embed these data into the same latent space, using both datasets as reference datasets, and used fastMNN^20^ to correct for any batch effects among the individual pools for each experimental platform. We observed that all cell types separated well, with good mixing between data collected from each modality (Fig. 4b).

**Extended Data Fig. 4:**
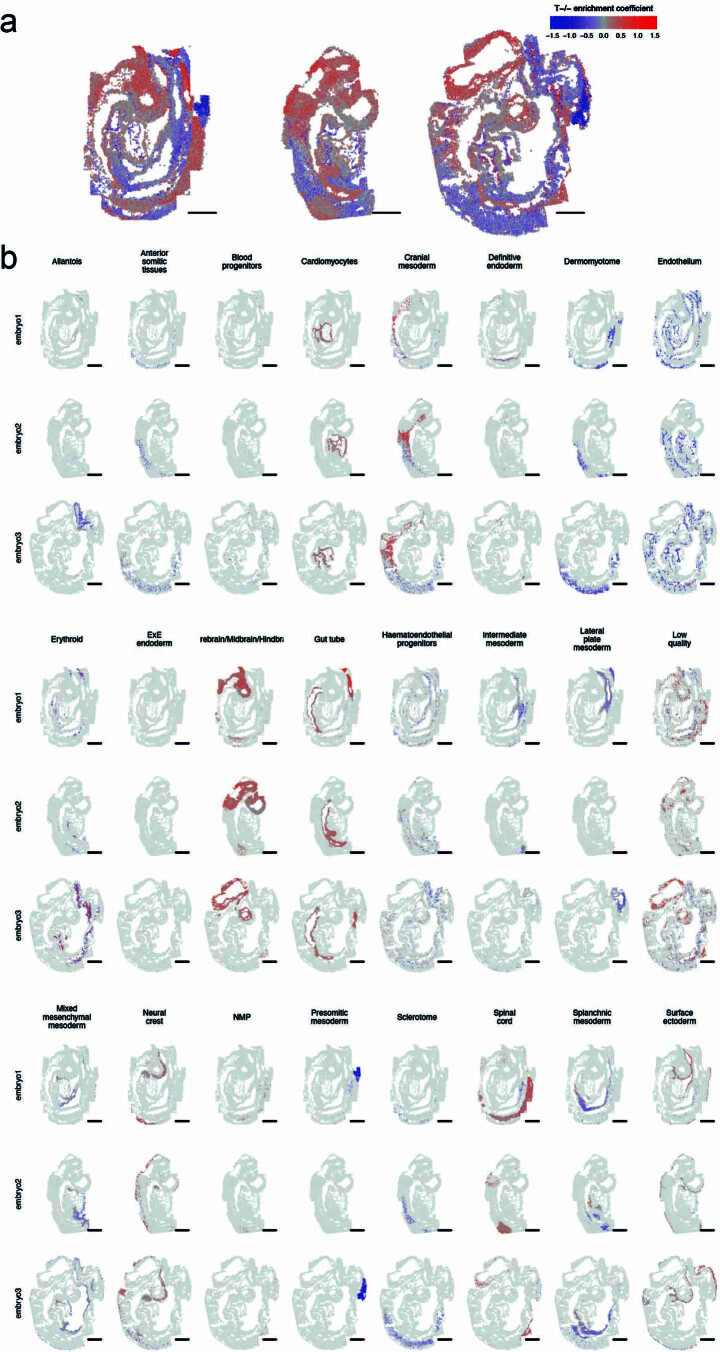
Local enrichment testing of seqFISH cells. **a**. Spatial coordinates plot of all seqFISH cells coloured by local coefficient value of T^−/−^ enrichment test. **b**. Spatial coordinates plots of all seqFISH cells, split by cell type (columns) and embryos (rows), where selected cells are coloured by local coefficient value of T^−/−^ enrichment test.

Given this joint embedding, we next performed spatially resolved enrichment testing of the relative abundance of T^−/−^ cells across the common space, to discover whether there are regions within the embryo where the T^−/−^ cells are enriched or depleted—an analysis that is possible only with the StabMap embedding. To do this, we first identified, for each seqFISH cell in the joint embedding, the 1,000 nearest neighbor cells from the T^−/−^/WT and the control WT/WT chimera samples. Among these 1,000 nearest neighbor cells, we calculated the relative fraction of cells contributing to the td-tomato^+^ population for each biological replicate of the T^−/−^/WT and WT/WT samples. Subsequently, for each seqFISH cell, we used logistic regression to statistically assess whether there was a local enrichment or depletion of T^−/−^ cells (Methods), identifying 16,677 significant seqFISH cells (false discovery rate (FDR)-adjusted *P* values <0.05 out of a total of 57,536 seqFISH cells) (Fig. 4c and Extended Data Fig. 4a).

Upon examining the annotation of these cells, we found, consistent with previous analysis^32^, broad depletion of T^−/−^ cells among the presomitic mesoderm, dermomyotome and sclerotome alongside broad enrichment in neuromesodermal progenitors (NMPs) (Fig. 4d and Extended Data Fig. 4b). Intriguingly, we observed a heterogeneous distribution of local T^−/−^ enrichment in the splanchnic/pharyngeal mesoderm (42 cells displaying significant positive enrichment and 543 cells displaying significant negative enrichment (FDR-adjusted *P* value <0.05)), a cell type associated with tissues surrounding the forming gut. When we examined the physical locations of these cells, we observed an extremely strong concordance between the local T^−/−^ enrichment coefficient and the relative positioning of the cells along the anterior-to-posterior (AP) axis, as quantified using principal curves^33^ (Spearman correlation ranging between −0.26 and −0.68; Fig. 4e and Methods).

**Extended Data Fig. 5:**
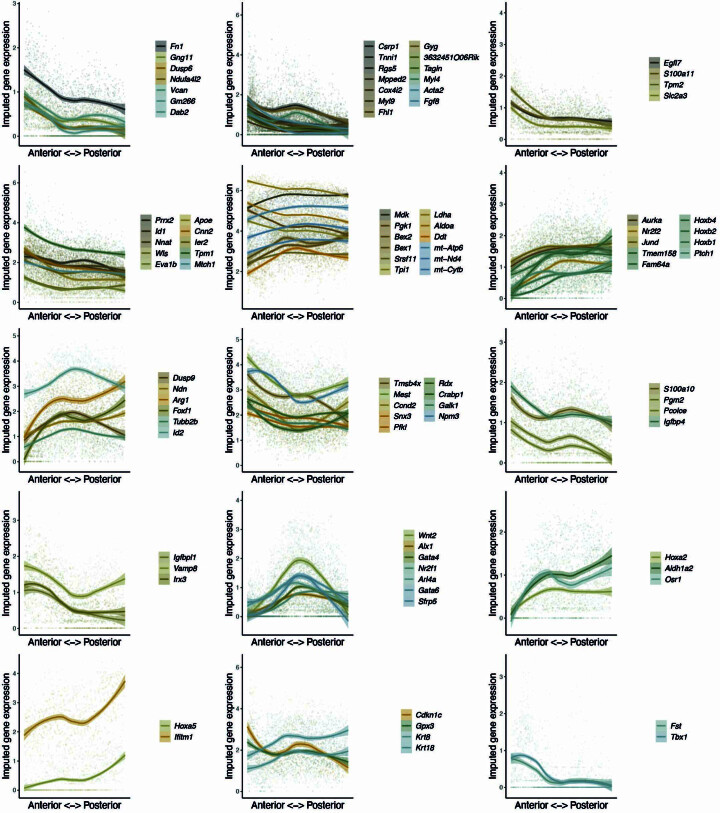
Significantly varying genes in spatial enrichment testing. Scatterplots and local mean expression ribbons of significantly varying genes (cubic splines likelihood ratio test FDR-adjusted P-values < 0.05), clustered using hierarchical clustering to show distinct patterns of expression along the AP axis in splanchnic mesoderm. Bands represent 95% confidence for loess smoothed fit.

We then used nonparametric cubic splines to identify imputed gene expression patterns that varied along the principal curve (Fig. 4f and Methods), and identified *Tbx1* and *Fgf8*, key genes regulating the development of anterior splanchnic mesoderm^34^ in the domain enriched for *T*^−/−^ cells. Conversely, markers of gut-associated splanchnic mesoderm *Foxf1 and Wnt2* (Fig. 4g)^35,36^ and of posterior mesoderm homeobox genes *Hoxb2* and *Hoxb4* (Extended Data Fig. 5) were enriched in the more posterior regions depleted in *T*^−/−^ cells.

Together, these observations suggest a broader role of Brachyury on regulating formation of posterior mesodermal tissues well beyond somitogenesis. In particular, this suggests that distinct domains of splanchnic mesoderm may also have distinct levels of dependency on Brachyury.

Our spatial mapping of the relative enrichment of T^−/−^ cells using StabMap provides a basis for mapping complex experimental data onto a spatial reference, thereby allowing us to draw these inferences without the need to perform spatial perturbation experiments.

## Discussion

In this paper, we have introduced StabMap, an approach to perform mosaic data integration for single cell data. StabMap accurately embeds single-cell data from multiple technology sources into the same low-dimensional coordinate space, using labeled or unlabeled single-cell data, and performs well even when some dataset pairs do not share any features. StabMap allows the use of one or more input datasets to be considered as references, and in general we suggest that datasets capturing potentially novel features, or a large amount of biological variation, be treated as reference datasets. In this vein, StabMap could be used to perform explicit mapping of query data onto a reference dataset, resulting in a joint embedding in the low-dimensional space as defined by the reference dataset alone.

A current limitation of StabMap is that all features from an experiment are considered together. However, for single-cell multiomics data an alternative would be to consider the different omics layers as individual data matrices, rather than to concatenate them into a large matrix^6^. This concatenation step corresponds to a naive example of vertical integration, where techniques such as feature standardization are employed to ensure comparability across different modalities measured in the same cell. StabMap could be extended to employ more sophisticated vertical integration techniques, for example, incorporating factors that describe variability across multiple layers, as implemented within MOFA^18^ or sharing information across multiple layers, as implemented within the weighted-nearest-neighbors framework^19^. In addition, more sophisticated modeling could be incorporated to extend StabMap beyond linear modeling. Such approaches would need to enable predictive mapping of new data through iterated projections, for example, support vector machines or elastic net regression.

A key advantage of StabMap is the ability to incorporate analytical features, which may exist for only a subset of datasets, in the data integration step. We have demonstrated this using the spatial seqFISH data integration by using the expression of each gene in the most proximal cells in physical space as a feature (something that cannot be captured in dissociated scRNA-seq data). Additionally, other bespoke features can be considered, such as local variance or local correlation values on spatial or trajectory-based data^37^, or cell-specific information such as lineage or clonal tracking information^38^. The ability to integrate data from such diverse sources offers the potential to extract biological insights by taking full advantage of diverse input datasets.

We envisage StabMap being used in a variety of contexts, especially as large-scale analysis of publicly available (and typically inconsistently processed datasets) becomes more widespread. Matching features between various datasets and ensuring a common data preprocessing pipeline is a serious hindrance for standard integration tools and can hinder the ability to draw biological insight. Consequently, StabMap could be employed to ensure that informative features are not lost purely due to practical challenges in preprocessing, enabling more comprehensive and complete downstream analysis.

## Methods

### MDT

The input to StabMap is a set of *s* appropriately scaled and normalized data matrices, ${\mathscr{D}}{\mathscr{=}}\{{{D}}_{1},{{D}}_{2},\ldots ,{{D}}_{{s}}\}$, not necessarily containing the same features, and optional discrete cell labels for any of the datasets. As an initial step, StabMap generates the corresponding MDT. The MDT is an undirected weighted network that contains *s* nodes, one corresponding to each data matrix, with edges being drawn between pairs of nodes for which the corresponding data matrices share at least one feature. The edges in the MDT are weighted according to the absolute number of common features between the two datasets. StabMap requires that the MDT be a connected network, that is, that there exists a path between any two nodes. Weighted shortest paths are calculated between any two given nodes in the MDT.

### The StabMap algorithm

At least one dataset must be considered as a reference dataset, with the option for multiple datasets to be considered as reference datasets. The output of StabMap is a common low-dimensional embedding with rows corresponding to all cells across all datasets, and columns corresponding to the sum of lower dimensions across the reference dataset(s). For a reference dataset *D*_*r*_, two matrices are extracted, first a scores matrix *S*_*r*_ (a cells × low-dimensions matrix) and a loadings matrix *A*_*r*_ (a features × low-dimensions matrix) such that ${S}_{{r}}={D}_{{r}}^{T}\times {A}_{{r}}$. If no cell labels are provided, principal components analysis (default 50 PCs) is used for estimation of *S*_*r*_ (as the PC scores) and *A*_*r*_ (the components loadings). Alternatively, if discrete cell labels are provided, linear discriminant analysis is used for estimation of *S*_*r*_ (as the linear discriminants for each class) and *A*_*r*_ (the feature discriminant loadings).

Then, for each of the *s* data matrices, score matrices ${{S}}_{1}^{{r}},{{S}}_{2}^{{r}},\ldots ,{{S}}_{{s}}^{{r}}$ are calculated in one of the following ways for data matrix *i*:

- If *i* = *r*, then the scores matrix *S*_*r*_ is returned, that is, ${{S}}_{{i}}^{{r}}={{S}}_{{r}}$.
- If *i* and *r* share an edge in the MDT, and all features in *A*_*r*_ are present in D_i_, then ${{S}}_{{i}}^{{r}}$ is directly calculated as the projected scores, that is ${S}_{i}^{r}={X}_{i}^{T}\times {A}_{r}$, where *X*_*i*_ is the appropriate submatrix of *D*_*i*_ to match the features in *A*_*r*_. If not all of the features in *A*_*r*_ are present in *D*_*i*_, then ${S}_{i}^{r}$ is estimated using multivariate linear regression on each column of *S*_*r*_ for dataset *D*_*r*_. Specifically, for column *j* of *S*_*r*_, we fit the model ${S}_{r}\left[\,j\right]={X}_{ < r,i > }\left[\,j\right]{\beta}_{ < r,i > }\left[\,j\right]+{{\epsilon }}$ where ${{X}}_{ < {r},{i} > }$ is the submatrix of *D*_*r*_ for features that are shared among *D*_*i*_ and *D*_*r*_, and *ϵ* is assumed to be normally distributed noise. ${B}_{ < r,i > }$ therefore is a matrix of fitted coefficients $\left({\hat{\beta }_{ < {r},{i} > ,1}},\ldots ,{\hat{\beta }_{ < {r},{i} > ,{j}}},\ldots \right)$ with rows corresponding to the shared features between *D*_*i*_ and *D*_*r*_ and columns corresponding to the columns of *S*_*r*_. The estimated score matrix for *i* is taken to be the predicted values of the multivariable linear model for dataset *D*_*i*_, and is calculated as ${{S}}_{{i}}^{{r}}={{X}}_{ < {i},{r} > }{{B}}_{ < {r},{i} > }$ where ${{X}}_{ < {i},{r} > }$ is the submatrix of *D*_*i*_ for features that are shared among *D*_*i*_ and *D*_*r*_.
- If *i* and *r* do not share an edge in the MDT, then ${S}_{i}^{r}$ is estimated using an iterative approach that exploits the shortest weighted path in the MDT. Starting from node *r*, for the next node along the path *p*, we calculate ${{S}}_{{p}}^{{r}}$ as described above. If the next node along the path is *i*, then we fit the model ${S}_{p}^{r}\left[j\right]={X}_{ < p,i > }\left[j\right]{\beta}_{ < p,i > }\left[j\right]+\epsilon$ where ${{X}}_{ < {p},{i} > }$ is the submatrix of *D*_*p*_ for features that are shared among *D*_*p*_ and *D*_*i*_ and ${{B}}_{ < {p},{i} > }$ is the matrix of fitted coefficients $\left({\hat{\beta}}_{< p,i > ,1},\ldots ,{\hat{\beta}}_{< p,i > ,j},\ldots \right)$. The estimated score matrix for *i* is then taken as the predicted values of this multivariable linear model for dataset *D*_*i*_, and is calculated as ${{S}}_{{i}}^{{r}}={{X}}_{ < {i},{p} > }{{B}}_{ < {p},{i} > }$. If instead, the next node along the path from *r* to *p* and eventually to *i* is some other node *q*, then this process of fitting a multivariable linear model and predicting on the new data is repeated until we calculate ${{S}}_{{i}}^{{r}}={{X}}_{ < {i},{q} > }{{B}}_{ < {w},{q} > }$, where *w* is the node previous to *q* along the path between *r* and *i*.

The estimated score matrices for each of the *s* datasets are then concatenated across rows to form the joint low-dimensional score where reference *r* is employed: ${{S}}^{{r}}=\left({{S}}_{1}^{{r}},{{S}}_{2}^{{r}},\ldots ,{{S}}_{{s}}^{{r}}\right)$, where *S*^*r*^ is a matrix with number of rows equal to the total number of cells across all *s* datasets and number of columns equal to the number of columns (selected features) in *S*^*r*^.

We believe StabMap’s improved performance over naive approaches can be explained by noting that the features that drive biological variation may either not be captured, or represent the dominant signal, in the shared feature space, and are therefore not prioritized when reducing dimensionality using PCA on the shared features. StabMap’s linear regression strategy estimates the linear combination of the shared features that best captures the (assumed to be) biological variation that is dominant in the full feature data.

### StabMap with multiple reference datasets

For the set of reference datasets *R* *=* {*D*_*j*_ s.t*. j* is in reference indices} ⊆ *D*, we calculate the corresponding set of joint low-dimensional scores as described above, *S* = {*S* ^*j*^ s.t. *j* is in reference indices}. We reweight each scores matrix *S* ^*j*^ according to the overall L1 norm of the matrix and a user-set weighting parameter ${{w}}_{{j}}\in \left[0,1\right]$ (by default set to 1),

$${{S}}^{{\,j}* }={{w}}_{{j}}\frac{{{S}}^{{\,j}}}{{\sum }_{{j}}\left|{{S}}^{{\,j}}\right|}.$$

The user-set weighting parameter *w*_*j*_ controls the magnitude of the score vectors for each reference dataset, and thus corresponds to the relative influence of the reference dataset on any magnitude-based downstream analysis (for example, calculation of Euclidean distances between cells). To generate common low-dimensional scores across all reference datasets, we concatenate the reweighted scores across columns to form the StabMap low-dimensional scores, ${S}=\left({{S}}^{{{\,j}}_{1}}{\rm{;}}{{S}}^{{{\,j}}_{2}}{\rm{;}}\ldots \right)$ for reference data indices *j*_1_, *j*_2_,…. *S* is a matrix with number of rows equal to the total number of cells across all *s* datasets, and number of columns equal to the total number of columns across the scores matrix for each reference dataset.

### StabMap computational speed

StabMap takes on the order of seconds to less than a minute for tens of thousands of cells on a standard MacBook. We observed StabMap taking on the order of 5–10 min running for 300,000 cells in our breast cancer analysis. We believe this speed can be attributed to several aspects of the software implementation. PCA is performed via the fast irlba algorithm, linear model fits are performed using the underlying R machinery via lm.fit, therefore reducing time and memory costs, and finally we retain the use of sparse matrix representation of data at every opportunity we can. While we use R’s native vectorization to speed up computation, one memory limitation at present is the need to convert to dense matrix representations for imputeEmbedding, this is due to the dependency of ‘abind’ package in R that works only for dense matrices. Future work could incorporate some sparse 3D array representation, thereby circumventing the need to convert data into dense matrices, or potentially to harness the capability of delayed matrix operations without needing to load data into memory. We find that runtime increases with the number of input datasets, as well as the proportion of datasets to be considered as references, as mapping across the MDT is repeated for each selected reference dataset.

### Downstream analysis with StabMap

#### Batch correction

While StabMap jointly embeds cells across multiple datasets into a common low-dimensional space, batch effects both within and among datasets can remain. Any existing batch correction algorithm that works on a low-dimensional matrix (for example, fastMNN^20^, scMerge^22^ and BBKNN^39^) can be employed to obtain batch-corrected StabMap embeddings. In the analyses presented in this manuscript we use fastMNN as downstream horizontal data integration. For the simulation presented in Fig. 2, we perform two additional horizontal data integrations using Harmony^26^ and Seurat^21^. For the latter case we treat the StabMap low-dimensional features as input features to Seurat, with parameters adjusted to not perform any feature selection or further dimensionality reduction.

#### Supervised and unsupervised learning

The batch-corrected StabMap embedding facilitates supervised learning tasks such as classification of discrete cell labels using any suitable method such as *k*-nearest neighbors, random forest and support vector machines, and regression using traditional linear models or support vector regression. Unsupervised learning tasks can be performed by clustering directly on the embedding (for example, *k*-means clustering) or by first estimating a cell–cell graph (for example, shared nearest neighbor or *k*-nearest neighbor graph) followed by graph-based clustering (for example, Louvain or Leiden graph clustering). Since one can use the embedding to estimate the cell–cell graph, additional bespoke single-cell analyses such as local differential abundance testing between experimental groups, such as that implemented in Milo^40^, can be employed.

#### Imputation of original features

We include an imputation implementation based on the StabMap low-dimensional embeddings to predict the full-feature matrices for all data, by extracting the set of *k* neighbors using Euclidean distance within the StabMap-projected space and returning the mean among the nearest neighbors. This is especially useful for projecting query data onto a reference space or for identifying informative features downstream of the data integration step.

### Mosaic data integration simulations

We used publicly available data to investigate the performance of StabMap and other methods, as described below.

#### PBMC 10x Multiome data

We used the SingleCellMultiModal R/Bioconductor package^41^ to download the ‘pbmc_10x’ dataset, containing gene expression counts matrix and read counts associated with chromatin peaks captured in the same set of cells. We normalized the gene expression values using logNormCounts^42^ in the scuttle package, and restricted further analysis to HVGs selected using the ModelGeneVar function in scran^43^. For the chromatin data modality we performed term frequency—inverse document frequency (TF-IDF) normalization according to the method described in ref. ^10^. We extracted peak annotation information using the MOFA2 R package tutorial^18^, including information on which genes’ promoters the chromatin peaks were associated with, if any. These promoter peaks were annotated as the associated gene name, so that the promoter peak features would match the RNA genes features.

To perform the mosaic data integration simulation with the PBMC 10x Multiome data, we ignored the matched structure between the RNA and chromatin modalities, and treated this data as if they belonged to two distinct datasets. We performed StabMap using both RNA and chromatin modalities as the reference datasets, and reweighted the embedding to give equal contribution for the two modalities. For assessing the cell type accuracy we used the RNA modality cells as labeled data, and predicted the cell types of the chromatin modality cells using *k*-nearest neighbors classification with *k* = 5.

#### Mouse Gastrulation Atlas scRNA-seq

We downloaded the counts data from Pijuan Sala et al. (2019)^1^ using the MouseGastrulationData R/Bioconductor package^44^ corresponding to E8.5, and normalized in the same way as the 10x Multiome PBMC data. Then, we split the dataset into four groups according to the four sequencing samples. For each randomly selected pair of sequencing samples, we artificially assigned one sequencing sample as the query dataset and kept one other sequencing sample intact as the reference dataset. Within each simulation round, we performed HVG selection from the reference dataset, and randomly selected 50, 100, 250, 500, 1,000, 2,000 and 5,000 genes to be kept for the query dataset.

We used StabMap to jointly embed the reference and query datasets into a common low-dimensional space by selecting the reference dataset as the sole reference, followed by batch correction using fastMNN. We also performed naive PCA, UINMF and MultiMAP for comparison. To assess performance, we calculated the mean accuracy of cell type classification of query cells using *k*-nearest neighbors with *k* = 5 for each method.

To assess the effect of downstream horizontal integration on embeddings using StabMap and naive PCA, we performed additional batch correction algorithms Harmony, fastMNN, and Seurat on the embeddings, as well as retaining uncorrected embeddings. We then calculated the difference in cell type accuracy between StabMap and naive PCA for each of the simulation scenarios and batch correction algorithms.

#### PBMC CyTOF data

We downloaded the PBMC CyTOF^27^ data using the HDCytoData^45^ package in Bioconductor. This dataset included two conditions of stimulated and unstimulated PBMCs from healthy individuals, of which we selected only unstimulated control cells for further analysis. From this data we extracted 24 protein features corresponding to biologically relevant signal.

#### PBMC ECCITE-seq data

We downloaded the PBMC ECCITE-seq data^28^ using the SingleCellMultiModal^41^ package in Bioconductor. This dataset included control and treated samples, from which we selected only control samples for further analysis. For these data, we extracted the single-cell RNA component and the cell surface ADT protein data.

#### Breast cancer IMC data

We downloaded the processed breast cancer IMC data^29^ using the Zenodo link provided in the publication. We selected only samples that corresponded to patients with positive estrogen receptor (ER) status and PAM50 classification of HER2, resulting in a set of 32,400 IMC-resolved cells, for which 37 protein features were profiled.

#### Breast cancer CITE-seq data

We downloaded the processed breast cancer CITE-seq data^30^ via GEO and the Broad Institute single-cell portal links provided in the publication. We selected a single patient sample, corresponding to an HER2-positive case. Then we combined the RNA and ADT modalities into a single data object using CiteFuse preprocessing tool^46^.

#### Breast cancer spatial transcriptomic data

We downloaded the processed breast cancer Xenium data^31^ on 3 November 2022 from the 10x Genomics website provided in the publication. We retained cells that captured at least 30 transcripts, and performed standardization using logNormCounts, resulting in a genes by cell expression matrix.

#### Comparison with other methods

##### UINMF

We used software version 0.5.0 of LIGER, which includes the UINMF implementation, and performed integration using defaults as suggested in the LIGER vignette. We used the counts matrix for input, as suggested in the vignette. We used the resulting 50-dimensional embedding for subsequent downstream analysis, and uniform manifold approximation and projection (UMAP) implemented in scater^42^ for visualization.

##### MultiMAP

We used the Python (version 3.8.10) package MultiMAP (version 0.0.1), and performed data integration using defaults as suggested by the MultiMAP tutorial website with equal weights for each dataset. The output of MultiMAP is a corrected graph representation, as well as a two-dimensional representation of the data. We used this two-dimensional representation for visualization and to perform downstream analysis tasks.

##### Naive PCA

To implement naive PCA, we first extracted the submatrices of datasets containing features that were common across all datasets. We then performed PCA using scran’s implementation with 50 principal components, followed by batch correction using MNN. We used the 50-dimensional representation for downstream analysis tasks, and UMAP to perform further dimensionality reduction to two dimensions for visualization.

##### Cobolt

We used the Python (version 3.8.10) package Cobolt (version 0.0.1), and performed data integration using defaults as suggested by the tutorial, with input data corresponding to the original counts for scRNA-seq gene expression and for ATAC detected open chromatin fragments. The output of Cobolt is a low-dimensional representation, which we further summarized using UMAP for visualization.

##### MultiVI

We used the Python (version 3.8.10) package scvi (version 0.16.4) and performed data integration using defaults as suggested by the package tutorial, with input datasets corresponding to original counts from scRNA-seq and ATAC seq multiomics, scRNA-seq and scATAC-seq. We extracted MultiVI latent space representation values, and performed UMAP for further visualization.

#### Evaluation

To evaluate the mosaic data integration simulations, we used three quantitative metrics.

##### Cell type classification accuracy

Given a joint embedding, we perform a simulation such that discrete class labels corresponding to cell types are artificially removed for a subset of the data. We then perform *k*-nearest neighbors classification (*k* = 5) to obtain the predicted class label for the artificially unlabeled data. The cell type classification accuracy is thus the proportion of cells for which the classification is correct compared to the true cell type label,

$$A=\frac{{\sum }_{i}I\{{C}_{i}^{{{\mathrm{pred}}}}={C}_{i}^{{{\mathrm{true}}}}\}}{{\sum }_{i}1}$$.

##### Jaccard similarity

For cell *i* in embedding *S* we have *l* positions for the *l* omics levels (for example, RNA and chromatin). We extract the sets of size *k* (default 100) containing the nearest cells of the same omics layer, that is, *N*_*il*_ = {set of neighbors of omics layer *l* s.t. rank (*D*(*S*_*il*_, *S*_*jl*_)) ≤ *k* where *D*(*a*,*b*) is the Euclidean distance of vectors *a* and *b*. The Jaccard similarity is thus

$${{J}}_{{i}}=\text{Jaccard}\left({{N}}_{{i}1},{{N}}_{{i}2}\right)=\frac{\left|{{N}}_{{i}1}\cap {{N}}_{{i}2}\right|}{\left|{{N}}_{{i}1}\cup {{N}}_{{i}2}\right|}$$.

Larger values of *J*_*i*_ correspond to larger overlap of neighbors between the two omics layers and are thus desired.

##### Number of nearest cells metric

Similar to the metric employed by Kriebel et al. and Jain et al.^11,12^, for cell *i* belonging to omics layer 1 (for example, RNA) in embedding *S*, we calculate the number of cells among omics layer 2 (for example, chromatin) that are nearer than cell *i* belonging to omics layer 2, ${{N}}_{{i}2}={\sum }_{{j}}{I}\{{D}\left({{S}}_{{i}1},{{S}}_{{j}2}\right)\le {D}\left({{S}}_{{i}1},{{S}}_{{i}2}\right)\}$.

We then extract the empirical cumulative distribution of nearest cells by calculating, for each integer *x*, the number of cells for which their number of nearest cells metric is at most this value, ${M}\left({x}\right)={\sum }_{{i}}{I}\{{{N}}_{{i}2}\le {x}\}$. Higher values of *M*(*x*) across all values of *x* are more desired.

#### Multi-hop mosaic data integration simulation

We used the PBMC 10x Multiome data to evaluate StabMap under the situation of multi-hop mosaic data integration. We downloaded and processed the data as described in the subsection above, with the exception that promoter peaks corresponding to specific genes were not matched to the associated genes. This resulted in a complete lack of overlap between features between the RNA and chromatin modalities.

To perform the simulation, we randomly allocated each cell into one of three classes: (1) RNA only, (2) chromatin only and (3) Multiome, with varying relative proportions of cells associated with the Multiome class. Cells within the RNA class had their chromatin information ignored, and cells within the chromatin class had their RNA information ignored, while cells within the Multiome class were left unchanged. We then used StabMap to integrate these three simulated datasets and generate a low-dimensional embedding for each simulation setting. Comparison with other methods is not possible since PCA, UINMF and MultiMAP require at least some overlapping features across all datasets.

To evaluate the multi-hop mosaic data integration simulation, we calculated the LISI^26^ using both modality and cell type as the grouping variables. Higher LISI values correspond to more local mixing of cells, and so relatively high values for modality and low values for cell type are desirable.

#### Multi-hop mosaic data integration of CyTOF, ECCITE-seq and 10x Multiome data

We used three data sources to examine StabMap’s capability of performing multi-hop mosaic data integration. We performed matching of protein IDs between the CyTOF and ECCITE-seq datasets, resulting in an overlap of seven proteins captured by each technology. For each dataset, we reassigned cell type labels to broad common cell types including B, CD4 T, CD8 T, dendritic cell (DC), MAIT T, monocyte, natural killer (NK) and surface cells. Then, we performed StabMap using three configurations. First, using the CyTOF dataset as the reference, with the underlying number of principal components set to 10 due to the limited number of proteins captured; second, using the 10x Multiome data as the reference; and third using both as references with equal weighting. In each case we performed downstream horizontal data integration using FastMNN. We visualized the resulting StabMap embeddings using UMAP. To assess the quality of each embedding, we used the LISI metric and examined the distribution of such values among the CyTOF and Multiome cells.

#### Multi-hop mosaic data integration of IMC, CITE-seq and 10x Genomics Xenium data

We used three data sources to examine StabMap’s ability to perform data integration, especially over multiple spatial omics technologies. We performed matching of protein ADT IDs between the IMC and CITE-seq datasets, resulting in 19 shared features. For the IMC and CITE-seq datasets, we reassigned cell type labels to broad common cell types including B cells, endothelial cells, epithelial cells, fibroblasts, myeloid cells, NK cells, plasmablasts, Panton-Valentine Leukocidin (PVL), T cells and tumor microenvironment (TME) cycling cells. Then we performed StabMap, selecting IMC and Xenium datasets as references, with 10 and 50 principal components respectively. Given the joint embedding extracted using StabMap, we then predicted epithelial cell class on the Xenium data, using the IMC-resolved cells as training data. Additionally, we performed feature imputation on the Xenium data, using the IMC-resolved data as training, using the imputeEmbedding function in the StabMap software. Finally, we predicted broad cell types on the Xenium data using the IMC-resolved cells as training data, and generated cell–cell contact maps (as previously described^7^) on two selected regions, corresponding to triple-positive receptor region, and an invasive region.

#### Simulation of multi-hop mosaic data integration using Mouse Gastrulation Data

To examine the capability of StabMap, we randomly selected cells from the Mouse Gastrulation Dataset described above, and split them into eight distinct datasets with varying numbers of total cells per dataset, *n* = 500, 1,000 and 2,000. Then, we retained varying numbers of features, *n* = 100, 200, 500 and 1,000 from among the HVGs such that there was approximately 50% overlap of features between datasets 1 and 2, 2 and 3, and so on. As a result, any one dataset only shared features with its neighboring dataset, representing an extreme task for multi-hop mosaic data integration. For the simulated datasets, we performed StabMap with dataset 1 selected as the reference dataset. To assess quality, we performed cell type classification (K-nearest neighbors (KNN) with *k* = 5) using dataset 1 as the training data and dataset 8 as the testing data, reporting the overall cell type classification accuracy as a measure of integration quality. We repeated the above simulation five times to obtain an overall mean accuracy with varying levels of number of cells and number of shared features.

#### Spatial mapping of mouse chimera data using StabMap

##### scRNA-seq data

We used the MouseGastrulationData R/Bioconductor package (Griffiths and Lun 2020)^44^ to download gene expression counts for the Mouse Gastrulation Atlas dataset, WT/WT control chimera dataset^1^, and T^−/−^/WT chimera dataset^32^, corresponding to E8.5. We combined the gene expression counts into a single dataset, then normalized and extracted HVGs using the same approach applied to the 10x Multiome PBMC data.

##### seqFISH data

We downloaded seqFISH-resolved gene expression log counts^7^ for spatially resolved cells of mouse embryos profiled at a similar developmental stage along with their corresponding spatial coordinates. We extracted novel features for each gene *g* and each cell *i* by calculating the mean expression value among the nearest cells in space, ${{x}}_{{gi}}^{* }=\frac{{\sum }_{{k}\in {{N}}_{{i}}}{{x}}_{{kj}}}{\left|{{N}}_{{i}}\right|}$, where ${N}$_*i*_ = {*k* s.t. *D*(*i*, *k*) ≤ 2, *i* ≠ *k*} is the set of cells that are at most two steps away from cell *i* in the spatial nearest neighbor network^7^. We then concatenated these novel features with the measured gene expression, before downstream integration with the dissociated scRNA-seq data.

##### Mosaic data integration and local enrichment testing

We used StabMap, parametrized with multiple reference datasets, to integrate the scRNA-seq and seqFISH data. We used PCA (default 50 PCs) to generate the low-dimensional scores for the scRNA-seq and seqFISH references, and reweighted each scores matrix using the default weighting parameter of 1. As a result, we obtained a 100-dimensional StabMap low-dimensional scores matrix. We then corrected for any remaining batch differences using fastMNN, where batches reflect technical groups from each dataset.

To calculate whether T^−/−^ cells were enriched in a neighborhood around each seqFISH cell, we performed logistic regression. Specifically, for each spatially resolved (seqFISH) cell, in the joint embedding we extracted its 1,000 nearest neighbors from each chimera dataset (4 T^−/−^/WT samples and 3 WT/WT samples), and fit the model ${\rm{log }}\frac{{p}}{1-{p}}={{{\upbeta }}}_{0}+{{{\upbeta }}}_{1}{{x}}_{1}+{{{\upbeta }}}_{2}{{x}}_{2}$.

In this model, *p* is the vector of observed proportions of td-tomato^+^ cells for each chimera, *x*_1_ is a vector containing the total proportion of td-tomato^+^ cells belonging to a biological replicate, and *x*_2_ is a vector indicating whether a chimera is T^−/−^/WT or WT/WT. We extracted the estimated coefficient of interest, $\hat{{{{\hskip0.5pt\beta }}}_{2}}$, and associated *P* value for each spatially resolved cell using a likelihood ratio test, resulting in a local measure of enrichment or depletion of T^−/−^ cells for each seqFISH-profiled cell. We then used the method of Benjamini–Hochberg to calculate FDR-adjusted *P* values.

##### Mixed T^−/−^ enrichment in pharyngeal/splanchnic mesoderm

To examine the relationship between the estimated T^−/−^ enrichment coefficient and AP axis position in the splanchnic mesoderm, we fitted principal curve models, with four degrees of freedom, for each individual spatially resolved embryo with the spatial coordinates as the underlying data^33^. We used the principal curve fitted values to extract the AP ranking of cells along this axis, and then used this ranking to estimate a locally smoothed T^−/−^ enrichment coefficient along the AP axis.

To assess gene expression changes along the AP axis as T^−/−^ cells move from being enriched to being depleted, we selected an equal number of cells anterior and posterior to the position where the smoothed T^−/−^ enrichment coefficient is zero, and performed differential gene expression analysis using imputed gene expression values. Imputed gene expression was quantified for each spatially resolved cell using the mean gene expression value of the nearest five Mouse Gastrulation Atlas cells in the StabMap low-dimensional space. Gene expression changes along the AP axis were assessed using a nonparametric cubic splines model with three degrees of freedom along with grouping variables for the individual embryos. Statistical significance was estimated using an *F*-test, with a null model of no splines effects, with empirical Bayes shrinkage using the limma framework, followed by adjustment for multiple testing. For statistically significant genes, we visualized gene expression along the AP axis using local loess smoothing and ribbon plotting for the local standard error.

### Reporting summary

Further information on research design is available in the Nature Portfolio Reporting Summary linked to this article.

## Online content

Any methods, additional references, Nature Portfolio reporting summaries, source data, extended data, supplementary information, acknowledgements, peer review information; details of author contributions and competing interests; and statements of data and code availability are available at 10.1038/s41587-023-01766-z.

### Supplementary information

Reporting Summary

## Data Availability

This study used publicly available data. The PBMC 10x Multiome, CyTOF, ECCITE-seq and mouse embryo scRNA-seq data were accessed via Bioconductor (version 3.13) ExperimentHub packages MouseGastrulationData (version 1.6.0), SingleCellMultiModal (version 1.4.0) and HDCytoData (version 1.14.0). The breast cancer IMC data were accessed via Zenodo (https://zenodo.org/record/6036188#.Y2Cu8exBxqs), the breast cancer CITE-seq accessed via GEO (accession GSE176078) and Broad Institute single-cell portal for protein ADT information (https://singlecell.broadinstitute.org/single_cell/study/SCP1039), and the breast cancer 10x Genomics Xenium data accessed via the 10x Genomics website (https://www.10xgenomics.com/products/xenium-in-situ/preview-dataset-human-breast) on 3 November 2022. The processed mouse embryo seqFISH data were accessed online via the web portal https://marionilab.cruk.cam.ac.uk/SpatialMouseAtlas/.

## Code availability

All analyses were performed in R (version 4.2.1). The StabMap software is available as an R package at https://github.com/MarioniLab/StabMap. Scripts for analysis and figure panels in this manuscript are available at https://github.com/MarioniLab/StabMap2021.

## References

[1] Pijuan-Sala B (2019). A single-cell molecular map of mouse gastrulation and early organogenesis. Nature.

[2] HuBMAP Consortium. (2019). The human body at cellular resolution: the NIH Human Biomolecular Atlas Program. Nature.

[3] Stoeckius M (2017). Simultaneous epitope and transcriptome measurement in single cells. Nat. Methods.

[4] Ma S (2020). Chromatin potential identified by shared single-cell profiling of RNA and chromatin. Cell.

[5] Luecken MD, Büttner M, Chaichoompu K (2022). Benchmarking atlas-level data integration in single-cell genomics. Nat. Methods..

[6] Argelaguet R, Cuomo ASE, Stegle O, Marioni JC (2021). Computational principles and challenges in single-cell data integration. Nat. Biotechnol..

[7] Lohoff, T. et al. Integration of spatial and single-cell transcriptomic data elucidates mouse organogenesis. *Nat. Biotechnol.*10.1038/s41587-021-01006-2 (2021). 10.1038/s41587-021-01006-2 PMC8763645 34489600

[8] Forcato M, Romano O, Bicciato S (2021). Computational methods for the integrative analysis of single-cell data. Brief. Bioinform..

[9] Lähnemann D (2020). Eleven grand challenges in single-cell data science. Genome Biol..

[10] Stuart T (2019). Comprehensive integration of single-cell data. Cell.

[11] Kriebel AR, Welch JD (2022). UINMF performs mosaic integration of single-cell multi-omic datasets using nonnegative matrix factorization. Nat. Commun..

[12] Jain MS, Polanski K, Conde CD (2021). MultiMAP: dimensionality reduction and integration of multimodal data. Genome Biol..

[13] Gong B, Zhou Y, Purdom E (2021). Cobolt: integrative analysis of multimodal single-cell sequencing data. Genome Biol..

[14] Ashuach, T., Gabitto, M. I., Jordan, M. I. & Yosef, N. MultiVI: deep generative model for the integration of multi-modal data. Preprint at *bioRxiv*10.1101/2021.08.20.457057 (2021). 10.1038/s41592-023-01909-9 PMC10406609 37386189

[15] Luo C (2022). Single nucleus multi-omics identifies human cortical cell regulatory genome diversity. Cell Genom..

[16] Abdelaal T, Mourragui S, Mahfouz A, Reinders MJT (2020). SpaGE: spatial gene enhancement using scRNA-seq. Nucleic Acids Res..

[17] Biancalani T (2021). Deep learning and alignment of spatially resolved single-cell transcriptomes with Tangram. Nat. Methods.

[18] Argelaguet R (2020). MOFA+: a statistical framework for comprehensive integration of multi-modal single-cell data. Genome Biol..

[19] Hao Y (2021). Integrated analysis of multimodal single-cell data. Cell.

[20] Haghverdi L, Lun ATL, Morgan MD, Marioni JC (2018). Batch effects in single-cell RNA-sequencing data are corrected by matching mutual nearest neighbors. Nat. Biotechnol..

[21] Butler A, Hoffman P, Smibert P, Papalexi E, Satija R (2018). Integrating single-cell transcriptomic data across different conditions, technologies, and species. Nat. Biotechnol..

[22] Lin Y (2019). scMerge leverages factor analysis, stable expression, and pseudoreplication to merge multiple single-cell RNA-seq datasets. Proc. Natl Acad. Sci. USA.

[23] Shi, M., Annika, K. & Michael, P. Nuclei isolation from tissue for 10x Multiome v1. Preprint at *protocols.io*10.17504/protocols.io.bukqnuvw

[24] Shah S, Lubeck E, Zhou W, Cai L (2016). In situ transcription profiling of single cells reveals spatial organization of cells in the mouse hippocampus. Neuron.

[25] Chen KH, Boettiger AN, Moffitt JR, Wang S, Zhuang X (2015). RNA imaging. Spatially resolved, highly multiplexed RNA profiling in single cells. Science.

[26] Korsunsky I (2019). Fast, sensitive and accurate integration of single-cell data with Harmony. Nat. Methods.

[27] Bodenmiller B (2012). Multiplexed mass cytometry profiling of cellular states perturbed by small-molecule regulators. Nat. Biotechnol..

[28] Mimitou EP (2019). Multiplexed detection of proteins, transcriptomes, clonotypes and CRISPR perturbations in single cells. Nat. Methods.

[29] Danenberg E (2022). Breast tumor microenvironment structures are associated with genomic features and clinical outcome. Nat. Genet..

[30] Wu SZ (2021). A single-cell and spatially resolved atlas of human breast cancers. Nat. Genet..

[31] Janesick, A. et al. High resolution mapping of the breast cancer tumor microenvironment using integrated single cell, spatial and in situ analysis of FFPE tissue. Preprint at *bioRxiv*10.1101/2022.10.06.510405 (2022). 10.1038/s41467-023-43458-x PMC10730913 38114474

[32] Guibentif C (2021). Diverse routes toward early somites in the mouse embryo. Dev. Cell.

[33] Hastie T, Stuetzle W (1989). Principal curves. J. Am. Stat. Assoc..

[34] Zhang Z, Huynh T, Baldini A (2006). Mesodermal expression of Tbx1 is necessary and sufficient for pharyngeal arch and cardiac outflow tract development. Development.

[35] Ormestad M (2006). Foxf1 and Foxf2 control murine gut development by limiting mesenchymal Wnt signaling and promoting extracellular matrix production. Development.

[36] Ustiyan V (2018). FOXF1 transcription factor promotes lung morphogenesis by inducing cellular proliferation in fetal lung mesenchyme. Dev. Biol..

[37] Ghazanfar, S. et al. Investigating higher-order interactions in single-cell data with scHOT. *Nat. Methods*10.1038/s41592-020-0885-x (2020). 10.1038/s41592-020-0885-x PMC7610653 32661426

[38] Bowling S (2020). An engineered CRISPR–Cas9 mouse line for simultaneous readout of lineage histories and gene expression profiles in single cells. Cell.

[39] Polański K (2020). BBKNN: fast batch alignment of single cell transcriptomes. Bioinformatics.

[40] Dann, E., Henderson, N. C., Teichmann, S. A., Morgan, M. D. & Marioni, J. C. Differential abundance testing on single-cell data using k-nearest neighbor graphs. *Nat. Biotechnol.*10.1038/s41587-021-01033-z (2021). 10.1038/s41587-021-01033-z PMC7617075 34594043

[41] Eckenrode, K. B. et al. Curated single cell multimodal landmark datasets for R/Bioconductor. Preprint at *bioRxiv*10.1101/2021.10.27.466079 (2021). 10.1371/journal.pcbi.1011324 PMC10497156 37624866

[42] McCarthy DJ, Campbell KR, Lun ATL, Wills QF (2017). Scater: pre-processing, quality control, normalization and visualization of single-cell RNA-seq data in R. Bioinformatics.

[43] Lun, A. T. L., McCarthy, D. J. & Marioni, J. C. A step-by-step workflow for low-level analysis of single-cell RNA-seq data with Bioconductor. *F1000Research*10.12688/f1000research.9501.2 (2016). 10.12688/f1000research.9501.1 PMC5112579 27909575

[44] Griffiths, J. & Lun, A. MouseGastrulationData: Single-Cell -omics Data across Mouse Gastrulation and Early Organogenesis. R package version 1.14.0 (2023); 10.18129/B9.bioc.MouseGastrulationData

[45] Weber LM, Soneson C (2019). HDCytoData: collection of high-dimensional cytometry benchmark datasets in Bioconductor object formats. F1000Research.

[46] Kim HJ, Lin Y, Geddes TA, Yang JYH, Yang P (2020). CiteFuse enables multi-modal analysis of CITE-seq data. Bioinformatics.

